# Virtual classroom proficiency-based progression for robotic surgery training (VROBOT): a randomised, prospective, cross-over, effectiveness study

**DOI:** 10.1007/s11701-022-01467-w

**Published:** 2022-10-17

**Authors:** Arjun Nathan, Sonam Patel, Maria Georgi, Monty Fricker, Aqua Asif, Alexander Ng, William Mullins, Man Kien Hang, Alexander Light, Senthil Nathan, Nader Francis, John Kelly, Justin Collins, Ashwin Sridhar

**Affiliations:** 1 Division of Surgery and Interventional Sciences, University College London, Gower Street, London, WC1E 6BT UK; 2 Royal College of Surgeons of England, London, UK; 3 University College London Hospitals NHS Foundation Trust, London, UK; 4 University College London Medical School, London, UK; 5 Newcastle University, Newcastle, UK; 6 St George’s University Hospital, London, UK; 7 Department of Surgery and Cancer, Imperial College London, London, UK

**Keywords:** Robotic skills training, Virtual classroom, Proficiency-based progression, Surgical education

## Abstract

Robotic surgery training has lacked evidence-based standardisation. We aimed to determine the effectiveness of adjunctive interactive virtual classroom training (VCT) in concordance with the self-directed Fundamentals of Robotic Surgery (FRS) curriculum. The virtual classroom is comprised of a studio with multiple audio–visual inputs to which participants can connect remotely via the BARCO weConnect platform. Eleven novice surgical trainees were randomly allocated to two training groups (A and B). In week 1, both groups completed a robotic skills induction. In week 2, Group A received training with the FRS curriculum and adjunctive VCT; Group B only received access to the FRS curriculum. In week 3, the groups received the alternate intervention. The primary outcome was measured using the validated robotic-objective structured assessment of technical skills (R-OSAT) at the end of week 2 (time-point 1) and 3 (time-point 2). All participants completed the training curriculum and were included in the final analyses. At time-point 1, Group A achieved a statistically significant greater mean proficiency score compared to Group B (44.80 vs 35.33 points, *p* = 0.006). At time-point 2, there was no significant difference in mean proficiency score in Group A from time-point 1. In contrast, Group B, who received further adjunctive VCT showed significant improvement in mean proficiency by 9.67 points from time-point 1 (95% CI 5.18–14.15, *p* = 0.003). VCT is an effective, accessible training adjunct to self-directed robotic skills training. With the steep learning curve in robotic surgery training, VCT offers interactive, expert-led learning and can increase training effectiveness and accessibility.

## Introduction

Over the last decade, there has been a growing utilisation of robot-assisted surgery, with an eightfold increase relative to laparoscopic techniques for common surgical procedures [1]. Better surgical dexterity, precision, 3-D visualisation, and ergonomics have driven surgeons to adopt this novel technology to better facilitate minimally invasive surgery [2]. Surgical training has been hampered by the cancellations of surgical procedures associated with the COVID-19 pandemic and coordinated strategic planning is necessary to improve the effectiveness and scalability of training [3].

Urology has emerged at the forefront of robotic surgery adoption, with Robot-Assisted Radical Prostatectomy (RARP) accounting for 89% of radical prostatectomy procedures in England between 2018 and 2019 compared to 5% laparoscopic and 6% open [4]. RARP has shown improved perioperative and functional outcomes in comparison to these other approaches [5–7]. Similar efficacy and safety outcomes have also been reported across many surgical subspecialties, including General Surgery [8, 9].

For novice robotic surgeons, robot-assisted surgery comprises difficult technical skills that require specialist training with a steep learning curve. Robotic surgery training has lacked standardisation, and the current model mainly consists of apprenticeship learning with variability in trainer skill and teaching method [10]. Recently, training centres have introduced proficiency-based modules and curriculums to certify and develop the skills of novice robotic surgeons [11]. The proficiency-based progression (PBP) training approach uses objective outcome measures with benchmarks based on the median performance of experienced surgeons, which must be achieved before progressing the trainee to advanced training. This practice has shown to be superior to standard time-based training approaches and ensures optimum skill acquisition at course completion with long-term retention of skills [12, 13].

The Fundamentals of Robotic Surgery (FRS) is the current accredited proficiency-based robotic training curriculum [14]. A recent multi-institutional randomised-controlled trial comparing skills training using the FRS and locally available robotic skills curricula found that trainees performed tasks faster and with fewer errors using the FRS [15]. Despite its effectiveness in teaching basic robotic principles, the FRS curriculum is self-directed with no trainer interaction or real-time performance feedback and advice.

Virtual classroom training (VCT) is a novel training modality that enables the combination of computer-based learning, with concurrent expert instruction and live interactive feedback via synchronous video communication [16]. It has the potential to offer increased effectiveness and accessibility to robot-assisted surgical training programs, reduce costs, and facilitate large-scale teaching. We aimed to determine the effectiveness of the Fundamentals of Robotic Surgery (FRS) training curriculum with adjunctive VCT.

## Methodology

### Participants

Students enrolled onto the University College London (UCL) Master of Science programme in Surgery and Interventional Sciences in 2020–2021 were voluntarily invited to participate in the study. Data were collected between February and March 2021. Participants had a minimum of 1-year clinical experience and no prior experience in robotic surgery training or operating.

### Intervention

The VCT session was hosted by an expert, high-volume robotic surgeon in a studio based at UCL. Participants were able to connect remotely via the BARCO weConnect platform. The studio comprises a live feed of the participants in attendance and a dual camera set-up allowing students to view the instructors in both close-up and wide shot. Instructors utilised the WeConnect software’s features an interactive digital whiteboard. The instructor-to-student ratio was 1:12.

### Study protocol

This was a prospective cohort, cross-over, efficacy study. Eleven participants were allocated into two training groups. In week 1, both groups received a robotic skills induction. In week 2, Group A undertook the self-directed modules of the FRS curriculum on the Intuitive daVinci^®^ surgical skills simulator, alongside interactive VCT. However, Group B only received access to the FRS curriculum. In week 3, participants crossed over and received the alternate intervention. Group A only received access to the FRS curriculum. Group B accessed the FRS curriculum, alongside interactive VCT (Table 1). Technical skills covered included: system component overview, safe set-up of system components, camera control, instrument handling, transferring, and swapping, managing encountered errors during system use. Non-technical skills covered included: team communication for optimal docking and safe instrument exchange, bleeding management, cardiopulmonary resuscitation (CPR), management of recoverable and non-recoverable faults, and effective communication with bedside assistants. The training tasks included docking and instrument insertion, ring tower transfer, knot-tying, railroad track task, fourth arm cutting, puzzle piece dissection, and vessel energy dissection. Training tasks were completed using the daVinci Si backpack surgical system and continued to be conducted until expert-derived proficiency was achieved.

**Table 1:** Robotic training course structure

	Group A	Group B
Week 1	Induction	Induction
Week 2	FRS + virtual classroom	FRS (self-directed)
Week 3	FRS (self-directed)	FRS + virtual classroom

Assessments were carried out after week 2 and week 3

*FRS* Fundamentals of Robotic Surgery

The primary outcome measure was an objective performance score achieved at competency quantified by the Robotic-Objective Structured Assessment of Technical Skills (R-OSATS) score. Participants were required to complete three robotic exercises on synthetic tissue models with a maximum score of 20 per exercise (60 in total). The three robotic exercises were:

1. Needle driving, knot-tying, and continuous suturing over a linear incision
2. Excision of lesion and wound closure
3. Needle driving simulation of anastomosis of tubular structures.

Scores were based on task errors, efficiency, dexterity, and tissue handling, and a higher score indicated better proficiency [17]. Two independent expert robotic surgeons examined the exercises. R-OSATS performance scores were collected at two time-points: time-point 1 was post-intervention with FRS only or VCT in addition to FRS (end of week 1), and time-point 2 was post-cross-over of both groups (end of week 2).

All participants completed a five-point Likert-scale questionnaire pre- and post-course, to assess self-reported confidence in performing the robotic skill tasks and an assessment of participant experience of virtual classroom training, including accessibility and feasibility of virtual classroom teaching.

### Ethical approval

All participants gave fully informed consent to participate in this study. This study was approved by the University College London Research Ethics Committee, ID: 19,683/001.

### Statistical analysis

Statistical analysis was conducted in IBM SPSS Statistics 27 [18]. Statistical significance was denoted by a *p* value of < 0.05. Welch’s two-tailed independent samples *t* test was used to compare proficiency between intervention groups at the two time-points. Two-tailed dependent samples *t* test was used to compare proficiency within groups at the 2 time-points. Two-way mixed ANOVA was used to test for the presence of an interaction between time and intervention group with respect to their effect on proficiency. Inter-rater reliability (IRR) was quantified by an intra-class correlation coefficient (ICC), which was calculated using a two-way, mixed-effects, consistency model. The IRR value was interpreted using cut-offs proposed by Koo et al. [19]. Likert-scale data were summarised descriptively.

## Results

All 11 robotic surgical novices completed the FRS curriculum with virtual classroom training (VCT). Five participants were assigned to Group A and six participants to Group B. No participants were excluded from the study. All data collected from the 11 participants were included in the analyses.

### Proficiency

**Fig. 1:**
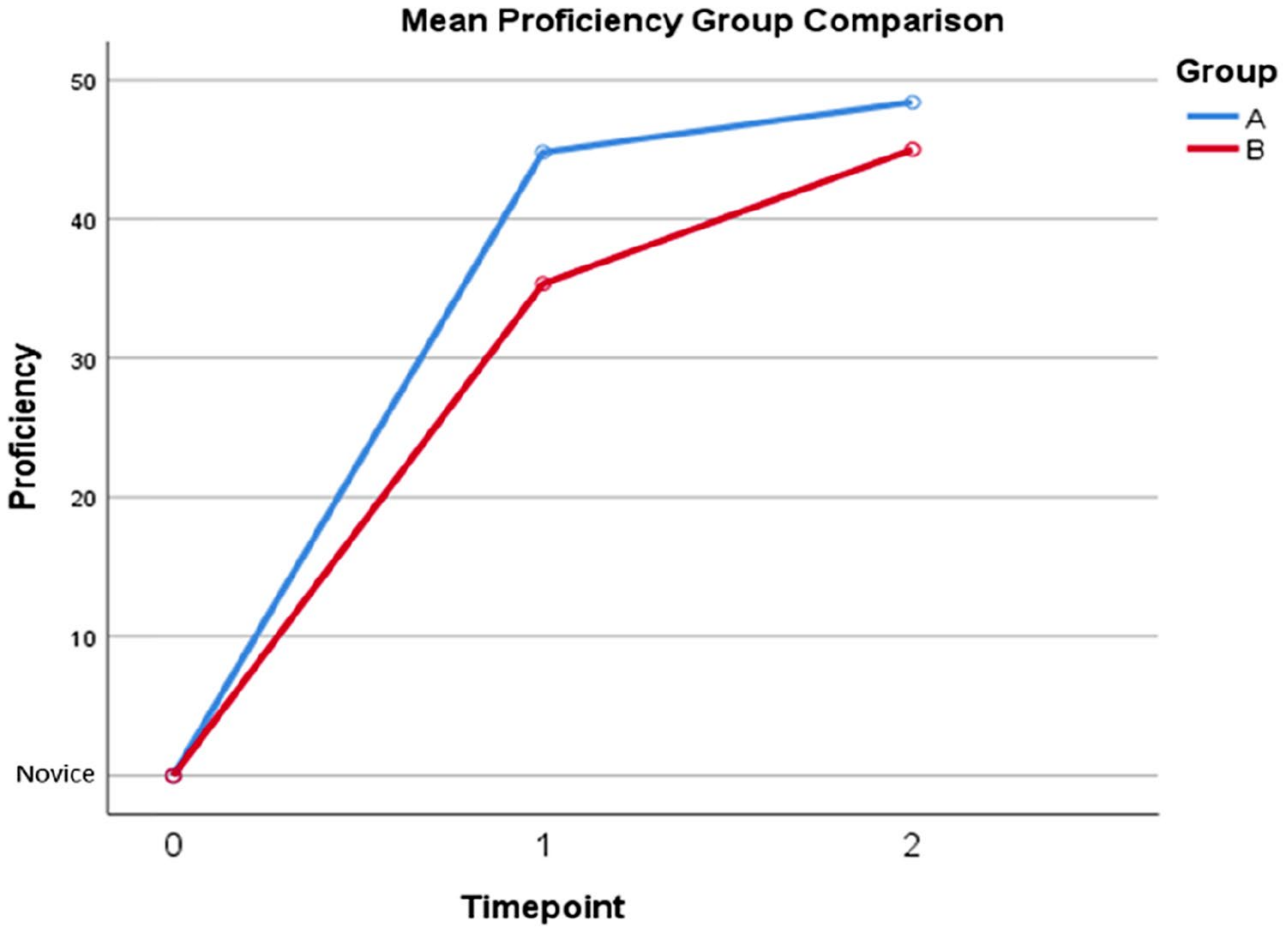
Mean proficiency assessment scores per group calculated at two time-points post-intervention with FRS and FRS + VCT. Group A had received training with FRS + VCT at time-point 1 and FRS alone at time-point 2. Group B had received training with FRS alone at time-point 1 and FRS + VCT at time-point 2

The mean proficiency scores for each group measured post-intervention at both assessment time-points are displayed in Fig. 1. On two-way ANOVA, both study groups demonstrated significantly improved proficiency over time (*F* [1, 9] = 19.41 points, *p* = 0.002). At time-point 1, Group A who received training with both the FRS and VCT sessions achieved a statistically significant greater mean proficiency score compared to Group B who received training under the FRS curriculum alone (44.80 vs 35.33 points, *p* = 0.006).

At time-point 2, both groups received the alternate intervention. There was no significant difference in mean proficiency score in Group A, the group that only had further self-directed learning, from time-point 1. In contrast, Group B, who received VCT, showed significant improvement in mean proficiency by 9.67 points from time-point 1 (95% CI 5.18–14.15, *p* = 0.003). No significant correlation was demonstrated over time between intervention group and proficiency scores (*F* [1, 9] = 4.06, *p* = 0.075). R-OSAT inter-rater reliability was quantified by an ICC value of 0.978 (95% CI 0.947, 0.991), indicating ‘excellent reliability.’

### Subjective confidence and perceptions

**Fig. 2:**
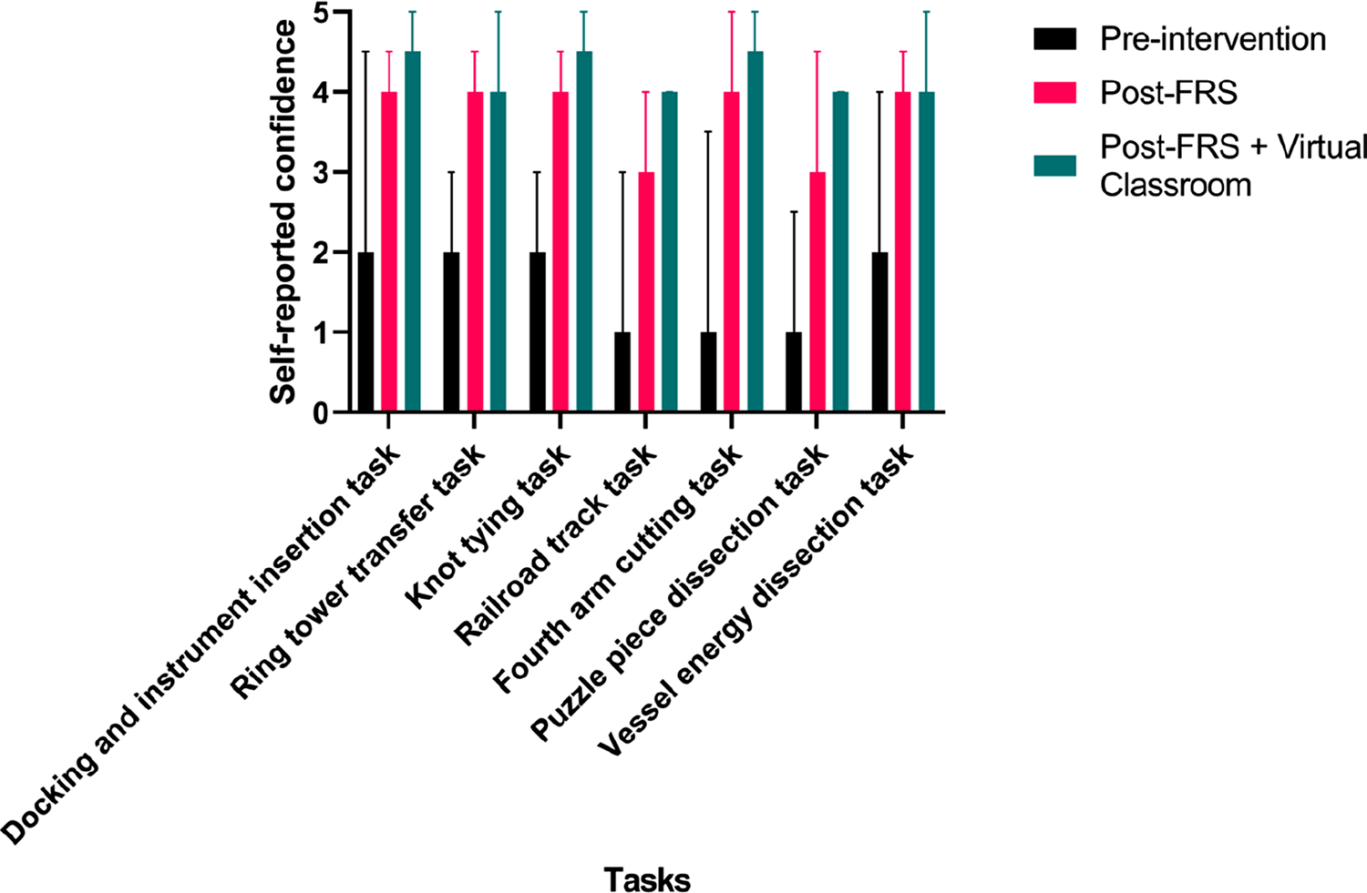
Self-reported confidence (median + IQR) in performing robotic tasks pre- and post-intervention with FRS and FRS + VCT

**Fig. 3:**
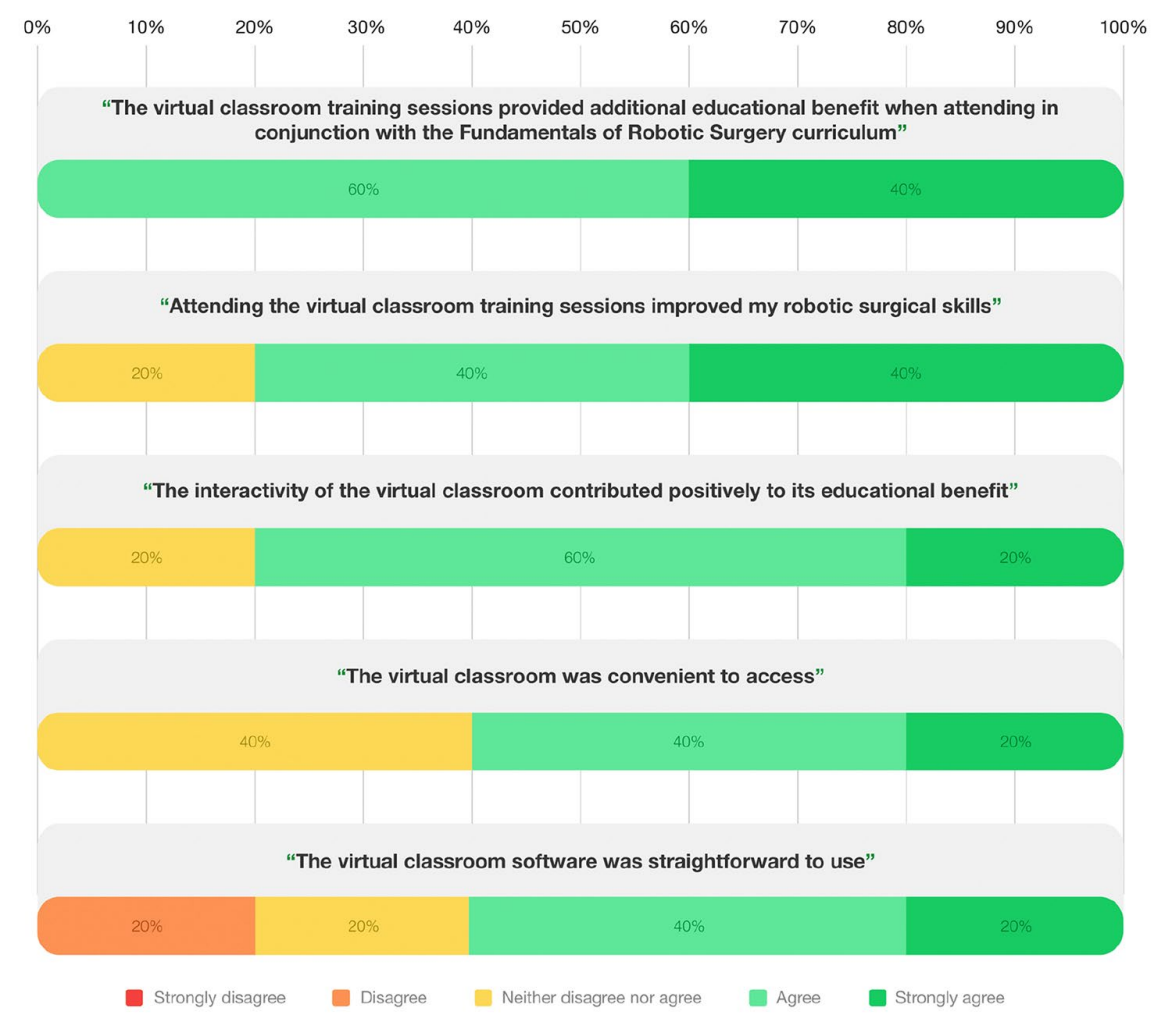
Survey response to statements regarding user experience and accessibility of virtual classroom training

Five of the eleven participants completed the subjective confidence rating questionnaires. The results of self-reported confidence in performing the assessed training tasks pre- and post-participation in the robotic skills training curriculum are displayed in Fig. 2. For all tasks, there was a significant improvement in subjective confidence post-robotic skills training with the FRS ± VCT. Five participants completed the five-point Likert-scale questionnaire evaluating trainee feedback of VCT. Participant’s perceptions of the virtual classroom training sessions are illustrated in Fig. 3. All (100%) participants agreed or strongly agreed that the VCT sessions provided additional educational benefit to the FRS curriculum, with four (80%) participants agreeing that the VCT sessions improved their technical robotic surgical skill. Four (80%) participants reported the beneficial impacts of interactivity within the VCT sessions.

## Discussion

Our study found that upon completion of the FRS course, all participants improved in proficiency; however, a significantly greater improvement in mean proficiency scores was seen if participants had undertaken VCT skills sessions in addition to the FRS curriculum.

Our findings are consistent with other feasibility studies demonstrating improvement in technical skill performance and confidence level by novice trainees following participation in a structured robotic surgery skills training curriculum [20–22]. However, many of these training curricula are directed to individual surgical specialties, focussing on institutional needs; a lack of standardisation remains in the robotic training pathway for novice surgeons. The FRS course was established to encompass all specialities performing robotic surgery and provides a generic introduction to core robotic surgical skills for novices that can be transferred to any robotic system. Our results suggest that the FRS curriculum is robust for teaching basic robotic skills and they support recent trials validating the use of the curriculum [15, 23].

Furthermore, all participants in our study retained proficiency of acquired skills over the duration of the training programme between the two assessment points, albeit this was over a period of 2 weeks and further assessment would be required to assess for longer term retention. The FRS curriculum integrates the principles of the PBP model [24], of which there has been widespread adoption in surgical training following its effectiveness in the aviation industry [11, 25]. A recent systematic review and meta-analysis found that PBP training significantly reduces the number of procedural errors by 60% compared to standard training [26]. The findings of Kho et al., who investigated 1-year skill retention following participation in a PBP robotic skills training curriculum, demonstrated that PBP courses yield a high level of retention of robotic surgical skills amongst trainees post-12.5 months of course completion [27]. These results suggest that such approaches to training can enhance skill durability, particularly important for trainees who do not have routine access to the surgical robot clinically.

The COVID-19 pandemic has propelled the adoption of virtual classroom technology in surgical education. The performance of the FRS and VCT group, and the cross-over group who undertook VCT corroborates the findings of Autry et al. who conducted a randomised-controlled trial investigating VCT for knot-tying. Over a 4-week period, 18 interns independently practiced knot-tying, nine of the interns were then randomly selected to receive three additional hours of VCT. Participants who attended VCT demonstrated greater proficiency improvement than the control group [28].

The FRS educational curriculum consists of online didactic modules and simulation-based self-practice. Supplementing the course with interactive virtual classroom teaching sessions offers an opportunity to receive mentorship from expert robotic surgeons. Our results highlight the beneficial effects of interactive sessions on objective performance scores and subjective confidence in performing robotic skill tasks. The importance of interactive learning has been acknowledged by teaching institutions, licensing bodies, and surgeons, especially when compared to didactic teaching. A prospective randomised-controlled trial by Tejos et al. demonstrated significantly inferior suturing skills training for medical students in the absence of concurrent peer or expert feedback [29]. Fayyadh et al. demonstrated that immediate auditory feedback is superior to other feedback types for surgical skills acquisition. Participants were stratified and randomly assigned to 5 experimental groups based on type (auditory versus visual) and timing (immediate versus delayed) of feedback [30]. Al-Jundi et al. compared distanced virtual feedback via video communication to face-to-face verbal feedback. It was concluded that the two modalities are quantitatively similar for basic surgical skills improvement among novice trainees [31].

The principal limitation of this study is its single institutional nature. The applicability to smaller centres, where continued access to VCT technology and robotic simulators may be challenging, is not known. Moreover, there was a small sample size (*n* = 11), which may underpower our analyses. Further investigation of performance outcomes in a larger cohort of trainees is required to be able to assess the value of virtual classroom teaching for robotic skills training. In addition, our study did not measure the cost-effectiveness of undertaking VCT sessions, including instructor time and virtual classroom technology cost. This is a factor that institutions will need to consider with implementing this approach to robotic skills training.

Robot-assisted surgery has gained momentum at an unprecedented pace, creating training challenges for novice surgical trainees. A recent pan-specialty trainee, cross-sectional study demonstrated that less than 12% of current surgical trainees reported having access to robotic surgery training opportunities [32]. To date, there have been numerous barriers identified for surgical trainees in accessing on-site practical robotic skills opportunities, particularly in relation to minimally invasive techniques. These include expensive and complex set-up of simulators, longer operation time extended by teaching, and reluctance of senior clinicians to hand over primary console control to trainees in theatre [33, 34]. These limitations have been further amplified during the pandemic [3]. There is evidence that the virtual classroom can improve access and efficiencies by increasing the number of delegates that can be trained in this educational setting, compared to face-to-face teaching [16]. Supplementing robotic skills training with virtual classroom teaching is a potential way to improve training and reduce the steep learning curve of robotic surgery to reach proficiency in robotic technical skills, in a scalable way that addresses current training needs.

## Conclusion

Interactive virtual classroom training is an effective training adjunct for robotic skills learning. We demonstrated higher mean proficiency scores attained upon completion of both the PBP-based FRS curriculum and VCT programme, compared to the FRS curriculum alone. We also found overall positive attitudes towards VCT sessions by robotic surgery novices.

## References

[1] Sheetz KH, Claflin J, Dimick JB (2020). Trends in the Adoption of Robotic Surgery for Common Surgical Procedures. JAMA Netw Open.

[2] Peters BS, Armijo PR, Krause C, Choudhury SA, Oleynikov D (2018). Review of emerging surgical robotic technology. Surg Endosc.

[3] Clements JM, Burke JR, Hope C, Nally DM, Doleman B, Giwa L, Griffiths G, Lund JN (2021). The quantitative impact of COVID-19 on surgical training in the United Kingdom. BJS Open.

[4] NationalProstateCancerAudit. NPCA Annual Report 2020 [Available from: https://www.npca.org.uk/content/uploads/2021/01/NPCA-Annual-Report-2020_Final_140121.pdf]

[5] Tewari A, Sooriakumaran P, Bloch DA, Seshadri-Kreaden U, Hebert AE, Wiklund P (2012). Positive surgical margin and perioperative complication rates of primary surgical treatments for prostate cancer: a systematic review and meta-analysis comparing retropubic, laparoscopic, and robotic prostatectomy. Eur Urol.

[6] Trabulsi EJ, Zola JC, Colon-Herdman A, Heckman JE, Gomella LG, Lallas CD (2011). Minimally invasive radical prostatectomy: transition from pure laparoscopic to robotic-assisted radical prostatectomy. Arch Esp Urol.

[7] Leow JJ, Chang SL, Meyer CP, Wang Y, Hanske J, Sammon JD (2016). Robot-assisted versus open radical prostatectomy: a contemporary analysis of an all-payer discharge database. Eur Urol.

[8] Roh CK, Choi S, Seo WJ, Cho M, Choi YY, Son T (2020). Comparison of surgical outcomes between integrated robotic and conventional laparoscopic surgery for distal gastrectomy: a propensity score matching analysis. Sci Rep.

[9] Li Y-P, Wang S-N, Lee K-T (2017). Robotic versus conventional laparoscopic cholecystectomy: a comparative study of medical resource utilization and clinical outcomes. Kaohsiung J Med Sci.

[10] Chen IHA, Ghazi A, Sridhar A, Stoyanov D, Slack M, Kelly JD (2020). Evolving robotic surgery training and improving patient safety, with the integration of novel technologies. World J Urol.

[11] Collins JW, Levy J, Stefanidis D (2019). Utilising the delphi process to develop a proficiency-based progression train-the-trainer course for robotic surgery training. Eur Urol.

[12] Ahlberg G, Enochsson L, Gallagher AG, Hedman L, Hogman C, McClusky DA (2007). Proficiency-based virtual reality training significantly reduces the error rate for residents during their first 10 laparoscopic cholecystectomies. Am J Surg.

[13] Mashaud LB, Castellvi AO, Hollett LA, Hogg DC, Tesfay ST, Scott DJ (2010). Two-year skill retention and certification exam performance after fundamentals of laparoscopic skills training and proficiency maintenance. Surgery.

[14] Smith R, Patel V, Satava R (2014). Fundamentals of robotic surgery: a course of basic robotic surgery skills based upon a 14-society consensus template of outcomes measures and curriculum development. Int J Med Robot Comput Assist Surg.

[15] Satava RM, Stefanidis D, Levy JS, Smith R, Martin JR, Monfared S (2020). Proving the effectiveness of the fundamentals of robotic surgery (frs) skills curriculum: a single-blinded, multispecialty Multi-institutional Randomized Control Trial. Ann Surg.

[16] Nathan A, Fricker M, Georgi M, Patel S, Hang MK, Asif A (2021). Virtual interactive surgical skills classroom: a parallel-group, non-inferiority, adjudicator-blinded, randomised controlled trial (VIRTUAL). J Surg Educ.

[17] Siddiqui NY, Galloway ML, Geller EJ, Green IC, Hur H-C, Langston K (2014). Validity and reliability of the robotic objective structured assessment of technical skills. Obstet Gynecol.

[18] [SPSS Statistics - Overview [Internet]. Ibm.com. 2021 [cited 29 January 2021]. Available from: https://www.ibm.com/uk-en/products/spss-statistics]

[19] Koo TK, Li MY (2016). A guideline of selecting and reporting intraclass correlation coefficients for reliability research. J Chiropr Med.

[20] Waters PS, Flynn J, Larach JT, Fernando D, Peacock O, Foster JD (2021). Fellowship training in robotic colorectal surgery within the current hospital setting: an achievable goal?. ANZ J Surg.

[21] Volpe A, Ahmed K, Dasgupta P, Ficarra V, Novara G, van der Poel H (2015). Pilot validation study of the European association of urology robotic training curriculum. Eur Urol.

[22] Arain NA, Dulan G, Hogg DC, Rege RV, Powers CE, Tesfay ST (2012). Comprehensive proficiency-based inanimate training for robotic surgery: reliability, feasibility, and educational benefit. Surg Endosc.

[23] Martin JR, Stefanidis D, Dorin RP, Goh AC, Satava RM, Levy JS (2020). Demonstrating the effectiveness of the fundamentals of robotic surgery (FRS) curriculum on the RobotiX mentor virtual reality simulation platform. J Robot Surg.

[24] Gallagher AG (2012). Metric-based simulation training to proficiency in medical education: what it is and how to do it. Ulster Med J.

[25] Collins JW, Wisz P (2019). Training in robotic surgery, replicating the airline industry How far have we come?. World J Urol.

[26] Mazzone E, Puliatti S, Amato M, Bunting B, Rocco B, Montorsi F (2021). A systematic review and meta-analysis on the impact of proficiency-based progression simulation training on performance outcomes. Ann Surg.

[27] Kho KA, Chen J, Hogg D, Sayers B, Scott DJ (2013). 1-Year skill retention following proficiency-based training for robotic surgery. J Minim Invasive Gynecol.

[28] Autry AM, Knight S, Lester F, Dubowitz G, Byamugisha J, Nsubuga Y (2013). Teaching surgical skills using video internet communication in a resource-limited setting. Obstet Gynecol.

[29] Tejos R, Crovari F, Achurra P, Avila R, Inzunza M, Jarry C (2021). Video-based guided simulation without peer or expert feedback is not enough: a randomized controlled trial of simulation-based training for medical students. World J Surg.

[30] Al Fayyadh MJ, Hassan RA, Tran ZK, Kempenich JW, Bunegin L, Dent DL (2017). Immediate auditory feedback is superior to other types of feedback for basic surgical skills acquisition. J Surg Educ.

[31] Al-Jundi W, Elsharif M, Anderson M, Chan P, Beard J, Nawaz S (2016). A randomized controlled trial to compare e-feedback versus standard face-to-face verbal feedback to improve the acquisition of procedural skill. J Surg Educ.

[32] Fleming CA, Ali O, Clements JM (2021). Surgical trainee experience and opinion of robotic surgery in surgical training and vision for the future: a snapshot study of pan-specialty surgical trainees. J Robot Surg.

[33] Turner SR, Mormando J, Park BJ, Huang J (2020). Attitudes of robotic surgery educators and learners: challenges, advantages, tips and tricks of teaching and learning robotic surgery. J Robot Surg.

[34] Farivar BS, Flannagan M, Leitman IM (2015). General surgery residents’ perception of robot-assisted procedures during surgical training. J Surg Educ.

